# Case series of hemostatic outcomes of defect closure using a novel anchor-pronged clip after gastric endoscopic submucosal dissection

**DOI:** 10.1016/j.igie.2025.10.012

**Published:** 2025-10-16

**Authors:** Kenichiro Okimoto, Tomoaki Matsumura, Shohei Mukai, Tsubasa Ishikawa, Akane Kurosugi, Michiko Sonoda, Tatsuya Kaneko, Yuki Ohta, Takashi Taida, Keisuke Matsusaka, Jun-ichiro Ikeda, Jun Kato

**Affiliations:** 1 Department of Gastroenterology, Graduate School of Medicine, Chiba University, Chiba, Japan; 2 Department of Diagnostic Pathology, Graduate School of Medicine, Chiba University, Chiba, Japan

## Abstract

**Background and Aims:**

Delayed bleeding (DB), occurring in 4.1% to 8.5% of gastric endoscopic submucosal dissection (ESD) cases, is one of the most common adverse events. The aim of this study was to evaluate whether defect closure using the novel anchor-pronged clip after gastric ESD contributes to the prevention of DB.

**Methods:**

A retrospective study at Chiba University Hospital (Chiba, Japan) included consecutive patients who underwent gastric ESD (August 2023–August 2024). The primary outcome was the rate of DB in the effective closure group, whereas the secondary outcomes included the effective closure rate and the full closure rate. Effective closure (≥50% of defect area) rate was defined as the proportion of study cases among all 127 cases. Full closure (≥90% of defect area) rate was defined as the proportion of study lesions among all 145 lesions during the period. The operators attempted to perform closure with the novel anchor-pronged clip for all cases. Among these, cases with effective closure were mainly analyzed. The first clip placed for defect closure was the novel anchor-pronged clip to achieve mucosa-to-mucosa or mucosa-to-muscle-layer closure. Additional clips could be either the same anchor-pronged clip or other typical through-the-scope clips. Clinical outcomes and adverse events including DB was calculated.

**Results:**

Out of 127 cases and 145 lesions, 111 cases and 124 lesions were included in the main analysis. The DB rate was 1.8%. The median diameters (interquartile range) (mm) of the macroscopic lesion and resected specimen were 10 (9-15) and 30 (25-35), respectively. The median closure time per lesion was 9 minutes. The effective closure and the full closure rate was 87.4% and 80.0%, respectively.

**Conclusions:**

Defect closure with the novel anchor-pronged clip after gastric ESD was associated with a high effective closure rate and a low DB rate (1.8%), suggesting that this method may help reduce the risk of DB.

## Introduction

Gastric cancer ranks as the fourth-most common cancer in men and the sixth in women worldwide, making it one of the major types of cancer.^1^ Endoscopic submucosal dissection (ESD) is a minimally invasive technique used to treat early gastric cancer with a high curative rate.^2^, ^3^, ^4^ However, one of the most adverse events associated with ESD is delayed bleeding (DB). DB specifically occurs in 4.1% to 8.5% of patients undergoing gastric ESD.^5^, ^6^, ^7^, ^8^, ^9^, ^10^

A review on post-ESD gastric closure including various types of closure with clipping and endoscopic hand-suturing reported that the DB rate was significantly lower in the closure group (5.4%) compared with the nonclosure group (12.4%).^11^ However, gastric defect closure is often challenging because of the influence of thick mucosa and muscle layers. Clip closure is considered one of the simplest and most time-efficient methods, and may offer several advantages. Although the utility of clip closure with the typical through-the-scope (TTS) clip in preventing DB after gastric ESD has been suggested, a previous study relied on selective inclusion of patients, thereby limiting the generalizability of its findings.^12^

A recent study reported the successful closure of postgastric ESD defect with the novel anchor-pronged clip (MANTIS Clip; Boston Scientific Japan, Tokyo, Japan).^13^, ^14^, ^15^ This novel anchor-pronged clip is reopenable and rotatable with an 11-mm opening width. However, these studies have been limited to case reports or technical notes. Although they demonstrated technical feasibility, they lacked a comprehensive evaluation of clinical outcomes, including DB rate. To address these gaps, we conducted a study to evaluate the clinical performance of the novel anchor-pronged clip in a consecutive patient cohort. This study aimed to evaluate whether defect closure using the novel anchor-pronged clip after gastric ESD contributes to the prevention of DB, based on a series of consecutive cases at our institution.

## Methods

### Study design

The novel anchor-pronged clip has been commercially available since March 2023. This retrospective study was conducted at Chiba University Hospital, Chiba, Japan. Consecutive patients who underwent gastric ESD between August 2023 and August 2024 were identified. The operator attempted to perform closure with the novel anchor-pronged clip for all cases (even if the lesion was located in the cardia or pylorus). Clinical outcomes and adverse events including DB were calculated. These factors were also compared between the effective closure group (included in the main analysis) and the closure failure group. Patients were included in the main analysis if effective closure was achieved. In cases with multiple lesions, only patients in whom all lesions met the effective closure criteria were included. Patients were excluded from the effective closure group under the following conditions: (1) closure failed for all lesions, (2) closure was successful for 1 lesion but failed for the other lesion, (3) closure was deemed technically impossible because of location and/or size, or (4) closure was not performed because of suspected risk of aspiration. Patients with these conditions were defined as the closure failure group.

### Outcomes

The primary outcome was the rate of DB in the effective closure group, whereas the secondary outcomes included the effective closure rate and the full closure rate.

### Details of ESD

Pre- and postoperative antithrombotic medications were administered according to the Japan Gastroenterological Endoscopy Society guidelines.^16^^,^^17^ ESD was performed with a conventional singe-channel endoscope with a water supply function (GIF-H290T; Olympus Medical Systems, Tokyo, Japan). After local injection of saline solution to confirm the location of the submucosa, a mixture of 0.4% sodium hyaluronate (MucoUp; Boston Scientific, Marlborough, Mass, USA) and normal saline in a 1:1 ratio was injected locally into the submucosa. DualKnife J (Olympus Medical Systems) was used for marking and cutting. An electrosurgical current was applied with the aid of an electrosurgical generator (VIO 3; Erbe Elektromedizin GmbH, Tübingen, Germany). If there were visible exposed vessels after ESD, thermal coagulation was performed using hemostatic forceps (RAICHO2 [KANEKA MEDIX, Osaka, Japan] or Coagrasper [Olympus Medical Systems]). Vonoprazan 20 mg was administered orally once a day starting on the day of ESD and continued generally for 28 days. The procedure time was defined as the duration from the start of the initial injection to the removal of the specimen.

### Defect closure after ESD

For the closure of the post-ESD defect, the first clip placed for defect closure was the novel anchor-pronged clip to achieve mucosa-to-mucosa or mucosa-to-muscle-layer closure (including cases where mucosa-to-submucosa closure is unavoidable) according to the defect size, thereby reducing the defect size. Defect closure was uniformly initiated with the use of an anchor-pronged clip, irrespective of defect size or location. If the size was large, the mucosa was grasped and traction was applied to the opposite side (Fig. 1, Videos 1 and 2 available online at www.igiejournal.org). After the size of the defect was reduced, additional clips aimed for full closure could be either the same anchor-pronged clip or other typical TTS clips (SureClip [Micro-Tech Co, Ltd, Nanjing, China] or EZ Clip [Olympus Medical Systems]) using mucosa-to-mucosa or mucosa-to-muscle-layer closure (including cases where mucosa-to-submucosa closure is unavoidable).^14^ In cases where a dead space was anticipated, mucosa-to-muscle-layer closure was continued, or mucosa-to-mucosa closure was performed using the novel anchor-pronged clips or typical TTS clips, incorporating the intervening muscle layer whenever possible to prevent its formation. If full closure was deemed unachievable, the procedure was concluded to the extent possible. All procedures were performed using a single-channel endoscope. Closure time was defined as the interval from inserting the first clip to completing placement of the final clip. Full closure was defined as the closure of ≥90% of the defect area. Effective closure was defined as the closure of ≥50% of the defect area. Failed closure was defined as the closure of <50% of the defect area. Dead space formation was defined as a lesion in which the operator determined that a clear dead space had formed in the submucosal layer after the completion of closure.

**Figure 1:**
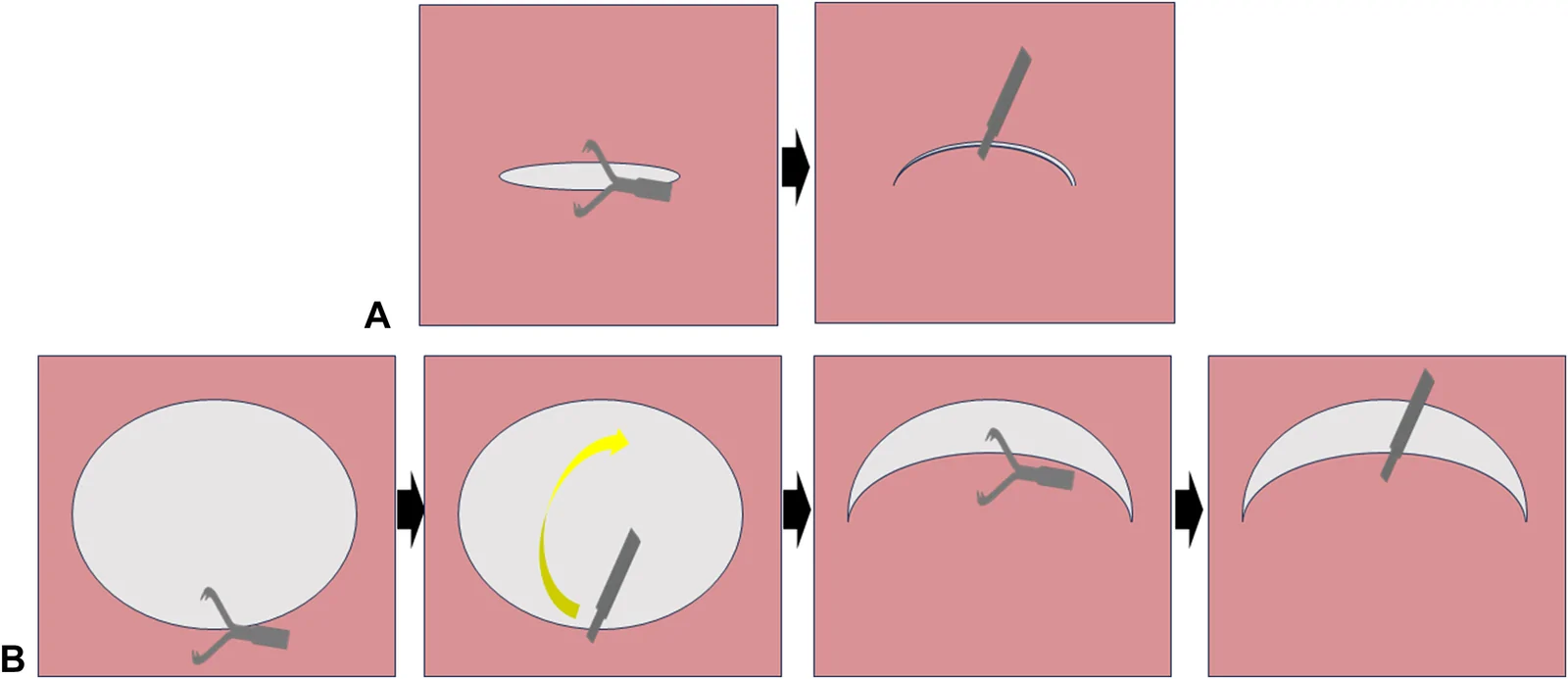
Initial clip placement for defect closure. The *pink area* represents the gastric mucosa, and the *gray area* represents the defect. **A,** Small defect: the first closure was performed using the novel anchor-pronged clip to achieve mucosa-to-mucosa closure. **B,** Large defect: the first closure was performed using the novel anchor-pronged clip to achieve mucosa-to-muscle-layer closure (including cases where mucosa-to-submucosa closure was unavoidable). Using the anchor-pronged clip, we grasped the mucosa and applied traction toward the opposite side; then the clip was reopened and closure was performed.

### Follow-up after ESD

ESD was performed during a 1-week hospitalization. When hematemesis or melena was observed, patients were instructed to contact the medical staff (primarily by telephone if the event occurred after discharge). In addition, patients were scheduled to return within 28 days after ESD for an explanation of the pathologic results, at which time the presence of DB was assessed. In this study, second-look endoscopy, including confirmation of defect closure, was not routinely performed except when DB was suspected. Subsequent follow-up endoscopy was usually conducted between 3 months and 1 year after the ESD. However, as follow-up was sometimes delegated to the referring hospital, detailed assessment of clip retention was not always possible.

### Full closure rate and effective closure rate

These factors were analyzed using an intention-to-treat analysis. The full closure rate was defined as the proportion of lesions that achieved full closure included in main analysis among all 145 lesions during the specified period. The effective closure rate was defined as the proportion of patients who achieved effective closure (accordingly, the proportion of the cases targeted in main analysis out of all 127 cases during the specified period).

### DB

DB was defined as hemorrhage with clinical symptoms and confirmed by emergency endoscopy within 28 days after ESD.^18^ Clinical symptoms were defined as hematemesis or melena, or a decrease in hemoglobin greater than 2 g/dL from the most recent laboratory test.^18^

### Statistical methods

Baseline data were presented as n (%) or medians with interquartile range (IQR). Continuous variables between the effective closure and failed closure groups were analyzed using the Mann-Whitney U test, whereas categorical variables were analyzed using the Fisher exact test when any expected cell count was ≤5, and otherwise using the χ^2^ test. The full closure rates according to tumor location and resected specimen diameter were analyzed using the χ^2^ test. All statistical analyses were performed using the Statistical Package for the Social Sciences software version 26 (IBM Corp, Armonk, NY, USA). *P* values of <.05 were considered statistically significant.

## Results

### Study flow

Figure 2 shows the study flow. During the specified period, 127 cases and 145 lesions underwent gastric ESD, and 111 cases and 124 lesions that achieved effective closure were targeted in the main analysis of the study. Sixteen cases with 21 lesions were failed closure.

**Figure 2:**
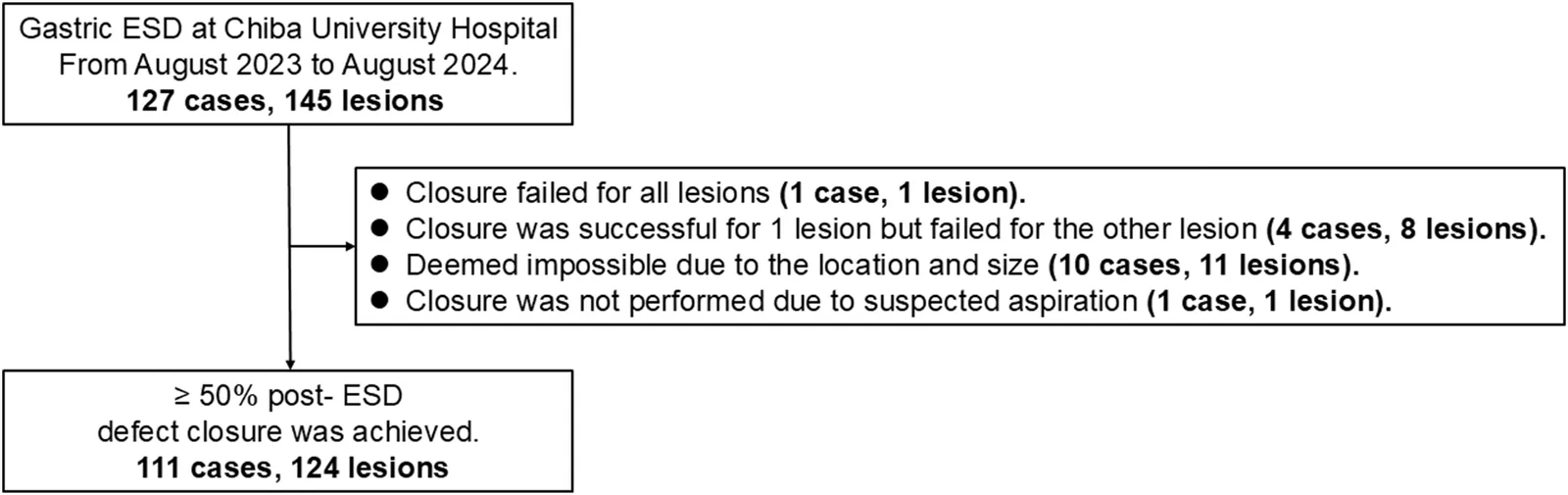
Study flow. *ESD*, Endoscopic submucosal dissection.

### Baseline features and clinical outcomes

In the effective closure group, there were 75 male and 36 female participants. The median age was 75 years (IQR 69-80). Antithrombotic therapy was administered in 31.5% of cases. No patients with cirrhosis or thrombocytopenia were identified in either group. Twelve patients in the effective closure group and 5 patients in the failed closure group had 2 lesions. One patient in the effective closure group had 3 lesions.

Both the median tumor diameter (10 mm, IQR 9-15) and the median resected specimen diameter (30 mm, IQR 25-35) were significantly smaller in the effective closure group than those in the failed closure group. The R0 resection rate (en bloc resection with free vertical and horizontal margins) was 89.5%. DB was observed in 2 cases (1.8%) in the effective closure group and tended to be lower than in the failed closure group (vs 6.3%, *P* = .335). No intraoperative or delayed perforations were observed (Table 1).

**Table 1:** Baseline features and clinical outcomes

Characteristics	Effective closure∗ (111 patients, 124 lesions)	Failed closure† (16 patients, 21 lesions)	*P* value
Age, years, median (IQR)	75 (69-80)	76 (71-83)	.467
Sex: male/female, n (%)	75 (67.6) / 36 (32.4)	7 (43.7) / 9 (56.3)	.114
Hemodialysis, n (%)	2 (1.8)	1 (6.3)	.323
Antithrombotic therapy, n (%)	35 (31.5)	5 (31.3)	1.000
Monotherapy	32 (28.8)	4 (25.0)	1.000
Aspirin	15 (13.5)	1 (6.3)	.692
Thienopyridine	6 (5.4)	2 (12.5)	.300
Cilostazol	4 (3.6)	1 (6.3)	1.000
Warfarin	0 (0)	0 (0)	1.000
DOACs	6 (5.4)	0 (0)	.596
Other antiplatelets	1 (0.9)	0 (0)	1.000
Dual therapy	3 (2.7)	1 (6.3)	1.000
Baseline features of the lesions			
Localization: upper/middle/lower, n (%)	18 (14.5) / 63 (50.8) / 43 (34.7)	6 (28.6) / 12 (57.1) / 3 (14.3)	.127
Macroscopic morphology: elevated/flat/depressed, n (%)	69 (55.6) / 5 (4.0) / 50 (40.4)	13 (61.9) / 2 (9.5) / 6 (28.6)	.715
Macroscopic tumor diameter, mm, median (IQR)	10 (9-15)	25 (10-50)	<.001
Resected specimen diameter, mm, median (IQR)	30 (25-35)	40 (30-61)	.004
Adenoma/IMC/SM invasive cancer, n (%)	16 (12.9) / 91 (73.4) / 17 (13.7)	0 (0) / 16 (76.2) / 5 (23.8)	.140
Clinical outcomes			
Procedure time, minutes, median (IQR)	50 (39-81)	64 (40-116)	.134
En bloc/R0‡ resection, n (%)	119 (96.0) / 111 (89.5)	20 (95.2) / 18 (85.7)	1.000/.705
Delayed bleeding, n (%)	2 (1.8)	1 (6.3)	.335
Intraoperative/delayed perforation, n (%)	0 (0) / 0 (0)	0 (0) / 0 (0)	1.000

*DOACs*, Direct-acting oral anticoagulants; *IMC*, intramucosal carcinoma; *IQR*, interquartile range; *SM*, submucosa.

∗ Closure of ≥50% of the defect area.

† Closure of <50% of the defect area.

‡ En bloc resection rate with free vertical and horizontal margins.

### Outcomes of defect closure after ESD

The median closure time per lesion was 9 minutes (IQR 2-35). In many cases, closure was completed within 10 minutes (66.1%), whereas only 15.3% of lesions required 15 minutes or longer. The effective closure rate was 87.4% (111/127), and the full closure rate was 80.0% (116/145). The full closure rates according to tumor location (upper, middle, and lower) were 70.8% (17/24), 77.3% (58/75), and 89.1% (41/46), respectively (*P* = .154), with a tendency toward a higher rate in the lower location. The full closure rates according to resected specimen diameter (mm) (≤30 vs >30) were 82.2% (74/90) and 76.4% (42/55), respectively (*P* = .416), with no significant difference. The median total number of clips per lesion was 7 (IQR 6-9), with the number of novel anchor-pronged clip per lesion being 1 (IQR 1-3) (Table2).

**Table 2:** Outcomes of defect closure after ESD (111 patients, 124 lesions)

Outcome	Value
Closure time per lesion∗, minutes, median (IQR)	9 (2-35)
Number of lesions with closure time ≤10 minutes, n (%)	82 (66.1)
Number of lesions with closure time ≥15 minutes, n (%)	19 (15.3)
Full closure rate†, n (%)	116 (80.0)
Effective closure rate‡, n (%)	111 (87.4)
Dead space formation rate, n (%)	18 (14.5)
Total number of clips per lesion, n, median (IQR)	7 (6-9)
Number of novel anchor-pronged clip per lesion, n, median (IQR)	1 (1-3)
Number of typical TTS clip per lesion, n, median (IQR)	5 (4-7)

*ESD*, Endoscopic submucosal dissection; *IQR*, interquartile range; *TTS*, through-the-scope.

∗ The interval from inserting the first clip to completing placement of the final clip.

† The proportion of lesions that achieved full closure (≥90% of the defect area) included in this study among all 145 lesions during the specified period.

‡ The proportion of patients who achieved effective closure (≥50% of the defect area). Accordingly, the proportion of the cases targeted in main analysis among all 127 cases during the specified period.

### Case presentation of DB

The first DB case is shown in Figure 3. The patient who continued aspirin therapy underwent ESD for 3 lesions, 1 of which (a 10-mm depressed lesion in the upper region) was suspected of DB. Although full closure was achieved in 3 minutes using 2 novel anchored clips and 3 typical TTS clips, melena was observed 7 days after ESD, and emergency endoscopy was performed. Soft coagulation was applied to the exposed vessel. The second DB case is shown in Figure 4 (10-mm depressed lesion in the lower region). The patient underwent ESD after continuing direct-acting oral anticoagulants (rivaroxaban) until the day before the procedure, with the medication withheld only on the day of ESD. Rivaroxaban was resumed on the day after ESD. Although full closure was achieved in 7 minutes using 1 novel anchored clip and 6 typical TTS clips, melena was observed 11 days after ESD, and emergency endoscopy was performed. After removal of the blood clot on the post-ESD ulcer, soft coagulation was performed on the exposed vessel. In both groups, patients were observed for DB until 28 days after ESD (21 days after emergency endoscopy in case 1 and 17 days in case 2), and no rebleeding was observed.

**Figure 3:**
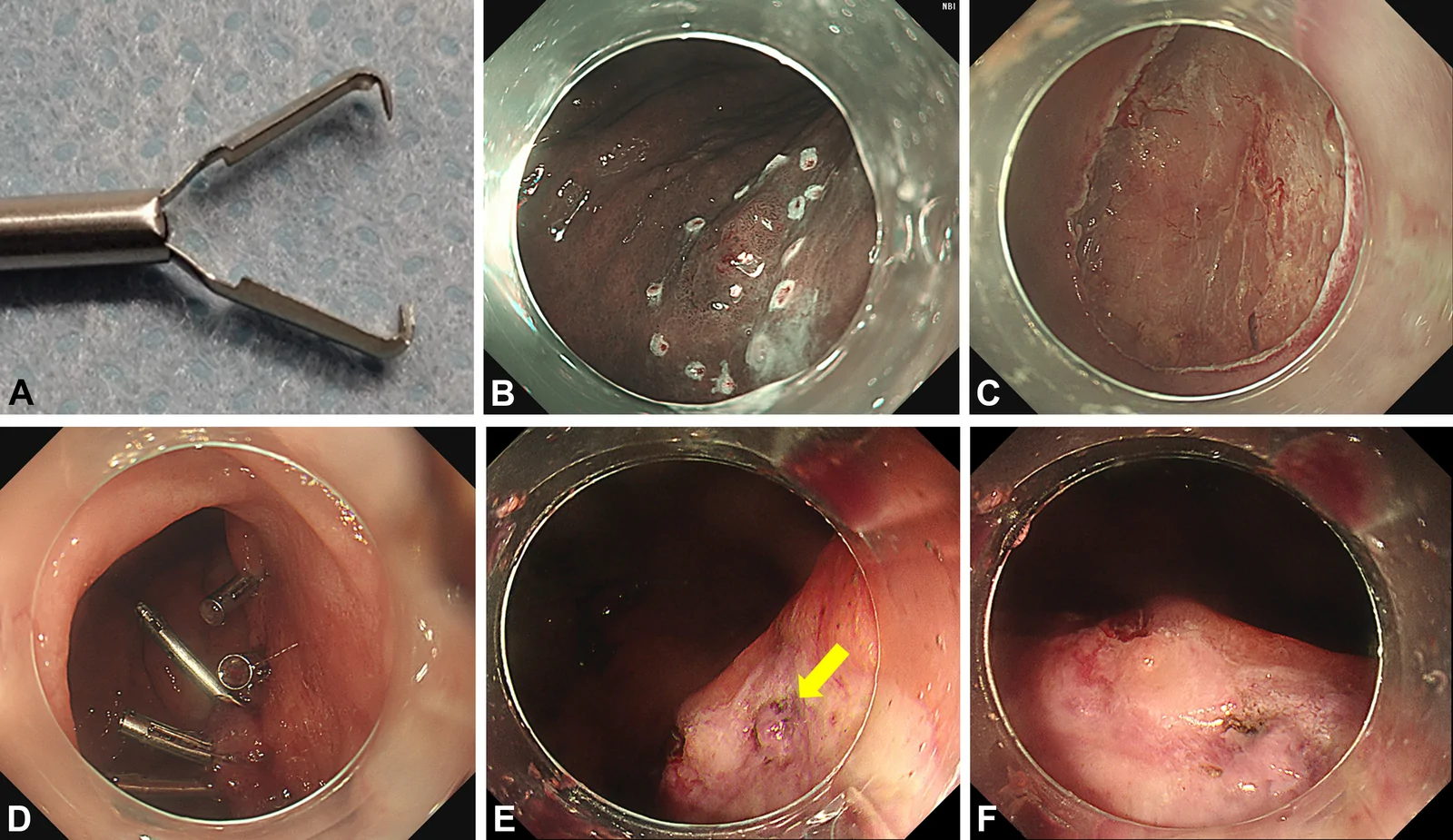
The first case of DB (10-mm depressed lesion in the upper region). **A,** The novel anchor-pronged clip that is reopenable and rotatable with an 11-mm opening width. **B,** After marking with narrow-band imaging. **C,** Post-ESD defect was about 40 mm. **D,** Full closure was achieved in 3 minutes using 2 anchor-pronged clips and 4 typical TTS clips. **E,** Melena was observed 7 days after ESD, and emergency endoscopy was performed. An exposed vessel was observed (*yellow arrow*). The clips had detached. **F,** Soft coagulation was performed on the exposed vessel. No rebleeding occurred. *DB*, Delayed bleeding; *ESD*, endoscopic submucosal dissection; *TTS*, through-the-scope.

**Figure 4:**
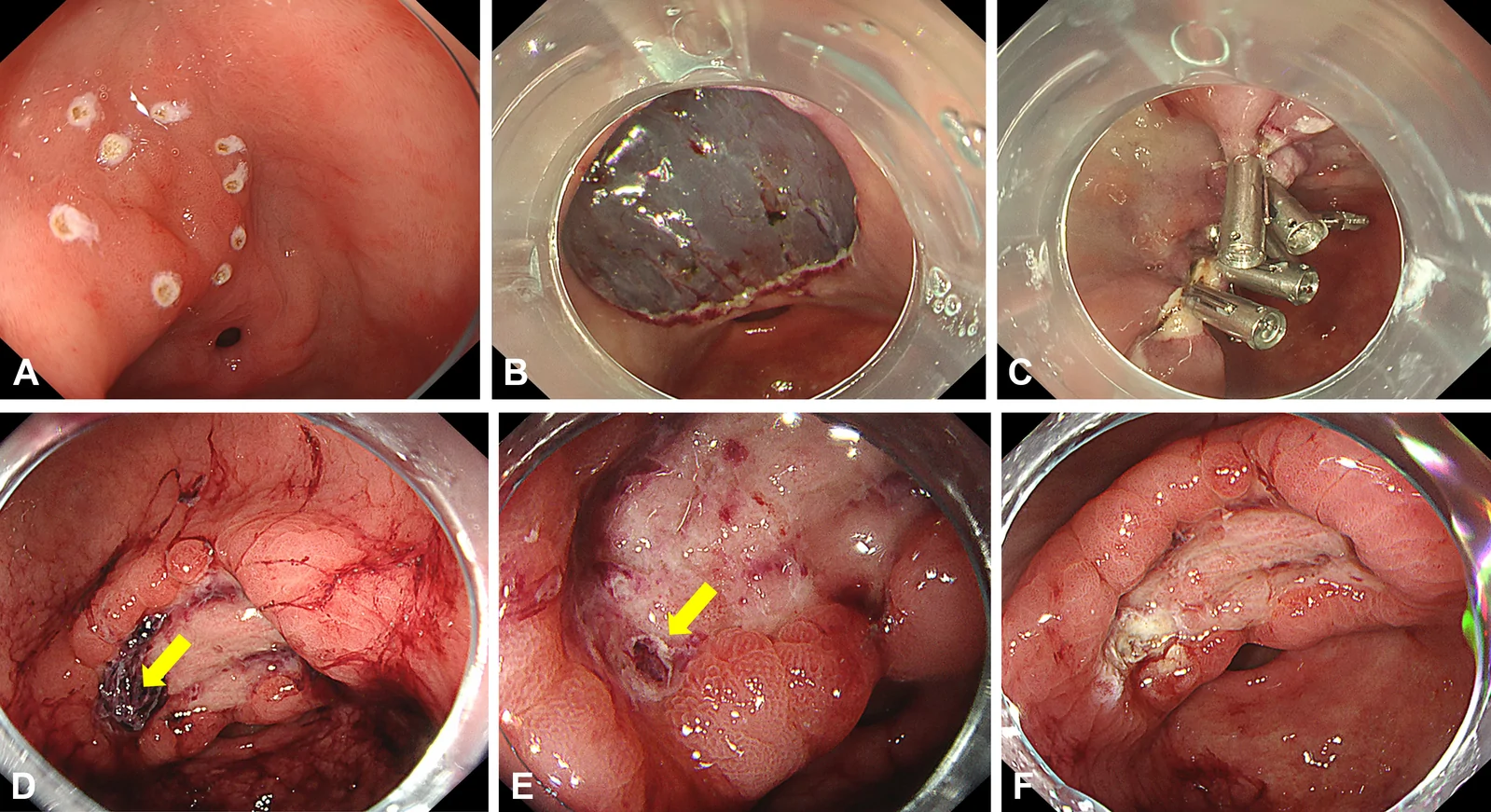
The second case of DB (10-mm depressed lesion in the lower region). **A,** After marking. **B,** Post-ESD defect was about 30 mm. **C,** Full closure was achieved in 7 minutes using 1 anchor-pronged clip and 6 typical TTS clips. **D,** Melena was observed 11 days after ESD, and emergency endoscopy was performed. A blood clot was attached to the post-ESD ulcer (*yellow arrow*). **E,** After removal of the blood clot, an exposed vessel was observed (*yellow arrow*). **F,** Soft coagulation was performed on the exposed vessel. No rebleeding occurred. *DB*, Delayed bleeding; *ESD*, endoscopic submucosal dissection; *TTS*, through-the-scope.

## Discussion

This study demonstrates the feasibility and favorable clinical outcomes of closure with the novel anchor-pronged clip after gastric ESD. To our knowledge, this is the initial report of a consecutive series of cases. Previous large-scale evaluations of the novel anchor-pronged clip for gastric ESD closure have been lacking, with prior reports primarily originating from Japan.^13^, ^14^, ^15^ The present study analyzed 111 cases with 124 lesions, representing the largest cohort to date in which a standardized closure method was applied, enabling a more robust assessment of clinical outcomes, including DB rate. By using the novel anchor-pronged clip, closure was feasible even for large post-ESD defects. Both the effective closure and the full closure rate were high, exceeding 80%. In addition, only 2 cases of DB were observed, resulting in a DB rate of 1.8%, which was lower compared with previous reports^5^, ^6^, ^7^, ^8^, ^9^, ^10^ and lower than the predicted rate of 5.1% based on the Bleeding after ESD Trend from Japan report.^18^ A recent retrospective study of 61 colorectal ESD cases demonstrated that mucosal defect closure using the novel anchor-pronged clip achieved a complete closure rate of 98% and a DB rate of 0%.^19^ Given the favorable outcomes in terms of DB as well as effective and full closure rates, our findings suggest that closure using the novel anchor-pronged clip may also be effective in the stomach, which has a thicker mucosal and muscle layer than the colorectum.

DB after gastric ESD can sometimes lead to hemodynamic shock, making its prevention highly desirable. Choi et al^12^ investigated postbleeding after gastric endoscopic mucosal resection/ESD using the typical TTS clip and reported a post-ESD bleeding rate of 3.3% with a tumor size of 16.7 mm. However, closure was performed in only 60 of 150 cases, leaving the true effectiveness unclear.^12^ At the very least, our method may result in a lower bleeding rate compared with the conventional method using the typical TTS clip. Our method is a modified approach based on the mucosa-submucosa clip closure method.^20^ The advantages of the closure method using the novel anchor-pronged clip include its ability to apply traction to the opposing mucosa, and its pronged shape enables grasping of the muscle layer. The high effective closure rate observed in our study may be attributed to a consistent closure protocol using the novel anchor-pronged clip as the initial anchor in a manner that achieves strong mucosa-mucosa, or mucosa-muscle closure appears to be the key to effective defect closure. As a result, a smaller dead space and more secure closure can be achieved even for a relatively large defect. When grasping the mucosa, submucosa, or muscle layer, it is crucial to perform full suction and close the clip slowly to minimize tissue damage and maximize the amount of tissue captured. Using a small number of the novel anchor-pronged clip in the initial stage of closure allows for efficient defect size reduction. In practice, the median number of the novel anchor-pronged clip used per lesion was 1.

Recently, new methods such as the reopenable-clip over-the-line method have been developed, enabling closure even for large gastric ESD defects, with a reported postbleeding rate of 1.8%, showing favorable outcomes.^21^ On the other hand, the average closure time has been reported as 35.6 minutes. Although our method has limitations in closing very large or complexly located defects, its major advantage is the short closure time, with a median of 9 minutes, and closure within 10 minutes was achieved in more than 60% of cases. The formation of a dead space in the submucosa raises concerns, including delayed defect healing, reduced closure strength due to the wide space, and difficulty in achieving endoscopic hemostasis in the event of bleeding from within the dead space. The method used in this study aimed to minimize dead space formation during closure, but approximately 15% of cases still resulted in dead space. However, at the very least, compared with mucosa-to-mucosa closure using the typical TTS clips, this method seemed to reduce dead space. Additionally, even if dead space forms, the mucosa remains closed, potentially protecting the defect from exposure to gastric acid and bile. Further investigation is needed to evaluate the relationship between dead space formation and postprocedural bleeding.

The limitations of this study are as follows: first, although consecutive cases were included, the study is single-center and retrospective with a small sample size. Second, there was no standardized evaluation period for clip retention. A prospective evaluation is necessary to accurately assess clip retention. Third, the closure was conducted by 4 main endoscopists (K.O., T.M., S.M., T.I.), which may affect the generalizability of the results. Fourth, because of the low incidence of DB, the comparison with the failed closure group did not reach statistical significance. Therefore, a prospective study is needed to confirm the reduction of DB suggested by the current study.

In conclusion, defect closure with the novel anchor-pronged clip after gastric ESD was associated with a high effective closure rate and a low DB rate (1.8%), suggesting that this method may help reduce the risk of DB.

## Ethics approval statement

This study was approved by the Medical Ethics Board of Chiba University Graduate School of Medicine (protocol HK202404-03) and was conducted in accordance with the Declaration of Helsinki.

## Patient consent

This was a retrospective cohort study, and written informed consent was not obtained. Informed consent was waived via the opt-out method.

## Disclosure

All authors disclosed no financial relationships.

## Supplementary data

Video 1 Cases of defect closure after gastric endoscopic submucosal dissection with the novel anchor-pronged clip: 25 mm elevated lesion in the Lower region of the stomach.

Video 2 Cases of defect closure after gastric endoscopic submucosal dissection with the novel anchor-pronged clip: 15 mm elevated lesion in the Upper region of the stomach.
